# Pegylated derivatives of recombinant human arginase (rhArg1) for sustained in vivo activity in cancer therapy: preparation, characterization and analysis of their pharmacodynamics *in vivo *and *in vitro *and action upon hepatocellular carcinoma cell (HCC)

**DOI:** 10.1186/1475-2867-9-9

**Published:** 2009-04-17

**Authors:** Sam-Mui Tsui, Wai-Man Lam, Tin-Lun Lam, Hiu-Chi Chong, Pui-Kin So, Sui-Yi Kwok, Simon Arnold, Paul Ning-Man Cheng, Denys N Wheatley, Wai-Hung Lo, Yun-Chung Leung

**Affiliations:** 1 Bio-Cancer Treatment International Limited, Bio-Informatics Centre, Hong Kong Science Park, Shatin, NT, Hong Kong; 2 Department of Applied Biology and Chemical Technology and Lo Ka Chung Centre for Natural Anti-Cancer Drug Development, The Hong Kong Polytechnic University, Hung Hom, Kowloon, Hong Kong; 3 BioMedES, Leggat House, Keithhall, Inverurie, Aberdeen AB51 0LX, UK

## Abstract

**Background:**

Protein used in medicine, e.g. interferon, are immunogenic and quickly broken down by the body. Pegylation is a recognized way of preserving their integrity and reducing immune reactions, and works well with enzymes used to degrade amino acids, a recent focus of attention in controlling cancer growth. Of the two arginine-degrading enzymes being explored clinically, arginine deiminase is a decidedly foreign mycoplasm-derived enzyme, whereas human arginase 1 is a native liver enzyme. Both have been pegylated, the former with adjuncts of 20 kD, the latter with 5 kD PEG. Pegylation is done by several different methods, not all of which are satisfactory or desirable.

**Methods:**

The preparation of novel polyethylene glycol (PEG) derivatives for modifying proteins is described, but directed specifically at pegylation of recombinant human arginase 1 (rhArg1). rhArg1 expressed in *Escherichia coli *was purified and coupled in various ways with 5 different PEG molecules to compare their protective properties and the residual enzyme activity, using hepatocellular cell lines both in vitro and in vivo.

**Results:**

Methoxypolyethylene glycol-succinimidyl propionate (mPEG-SPA 5,000) coupled with very high affinity under mild conditions. The resulting pegylated enzyme (rhArg1-peg_5,000 mw_) had up to 6 PEG chains of 5K length which not only protected it from degradation and any residual immunogenicity, but most importantly let it retain >90% of its native catalytic activity. It remained efficacious in depleting arginine in rats after a single ip injection of 1,500 U of the conjugate as the native enzyme, plasma arginine falling to >0.05 μM from ~170 μM within 20 min and lasting 6 days. The conjugate had almost the same efficacy as unpegylated rhArg1 on 2 cultured human liver cancer (HCC) cell lines. It was considerably more effective than 4 other pegylated conjugates prepared.

**Conclusion:**

Valuable data on the optimization of the pegylation procedure and choice of ligand that best stabilizes the enzyme arginase 1 are presented, a protocol that should equally fit many other enzymes and proteins. It is a long lasting arginine-depleting enzyme in vivo which will greatly improve its use in anti-cancer therapy.

## Background

Arginine degrading enzymes have been used to treat cancer for some time [1,2]. We have recently published findings with pegylated arginase both *in vitro *and *in vivo *[3,4]; a brief overview can be found in Cheng and Wheatley, 2007 [5] that show how effective pegylation can be in protecting even a native human enzyme from rapid elimination by one means and another from the bloodstream. Hepatocellular carcinoma (HCC) is a prime example of a tumour that should be amenable to treatment with this enzyme, since they have previously been considered auxotrophic for arginine because they do not express argininosuccinate synthetase (ASS; e.g. [6]). While the arginine-depleting enzyme arginine deiminase (ADI) has been used to treat ASS-deficient tumors [7], arginase can also be effective in treating ASS-positive tumors because it eliminates the constant recycling of citrulline in a manner that cannot be emulated by the former enzyme [4]. The latter report also recognised that another enzyme deficiency was key in many HCC cases, viz. ornithine transcarbamylase (OTC), enhancing the possibility of a good therapeutic response to arginase administration. This may open up exciting possibilities for the effective treatment of not only HCC, but probably a range of other tumours where OTC levels are also under investigation.

There is currently a groundswell in the exploration of arginine dependency of tumours of diverse types. Whatever the enzyme preference for use in clinical work (and one can also include, for example L-asparaginase [8] and L-methioninase [9]), it is of paramount importance that it is both safe and highly efficacious. Sometimes it is impossible to achieve this without compromising activity, and therefore if this can be avoided, improved preparations may well be obtained. *Arginase 1 *is a natural liver enzyme, but arginine deiminase is an enzyme expressed by mycoplasm that can be purified from the culture medium, or expressed as a recombinant form by transfection of *E. coli *with the ADI gene, as for arginase 1. Irrespective of which enzyme is chosen for use in cancer therapy, it would be of considerable value to standardise a procedure that produces the optimal pegylation for the protection of the enzyme against its rapid destruction in vivo while retaining maximal activity against its substrate, arginine in this case. It is also desirable that pegylation does not itself have any associated toxicity producing undesirable side effects. These are crucial matters that need to be investigated and resolved to general satisfaction before many more clinical studies are undertaken. We present data that establishes for arginase 1 a mode of pegylation that meets the required criteria, and which can be equally applicable to many other suitable proteins (i.e. with free ε amino groups).

Arginase is a urea cycle enzyme that catalyzes the hydrolysis of L-arginine to L-ornithine and urea in a divalent cation-dependent (Mn^2+^) reaction [10]. Recombinant human arginase I (rhArg1) has been successfully cloned and expressed in *Bacillus subtilis *[11] and *E. coli *[12]. However, the unacceptably short circulatory half-life of native arginase in the human body (t_1/2 _<30 min) makes it difficult to maintain a therapeutically effective (low) blood arginine concentration in patients without frequent administration of large doses that are – from an economic viewpoint – difficult to sustain with the present availability of the enzyme. The attachment of polymers to proteins, e.g. polyethylene glycol (PEG), is an established technique to enhance the therapeutic and biotechnological potential of numerous proteins [13-16]. Examples of protected proteins include: mPEG-IL-2 [17], mPEG-IFN-α [18], pegylated recombinant interferon alpha-2b, recombinant methioninase [9], pegylated arginine deiminase (ADI, another arginine-depleting enzyme; [7]), and pegylated bovine liver arginase [19,20]. In the last example, pegylated bovine liver arginase retained only ~65% of its original enzymatic activity, only ~53% of the free amino (mainly lysine) groups were conjugated with PEG molecules, and its circulatory life was <12 h [19].

Improved techniques of pegylating arginase not only enhance the half-life of arginase *in vivo*, but can quite profoundly reduce the immunogenicity of the protein, protecting it from degradation by directly masking surface antigens and sterically hindering interactions with antibodies [13]. While this is mandatory with foreign enzymes such as ADI [21], it is far less relevant where the native human enzyme is used. rhArg1 may, however, show some immunogenicity because of its expression in the 6×His-tagged form in bacteria, and also because its isolation and purification procedures do not ensure 100% recovery of its native form [11]. However, its increased size after pegylation reduces renal clearance, which also helps prolong its circulatory half-life [16].

**Figure 1:**
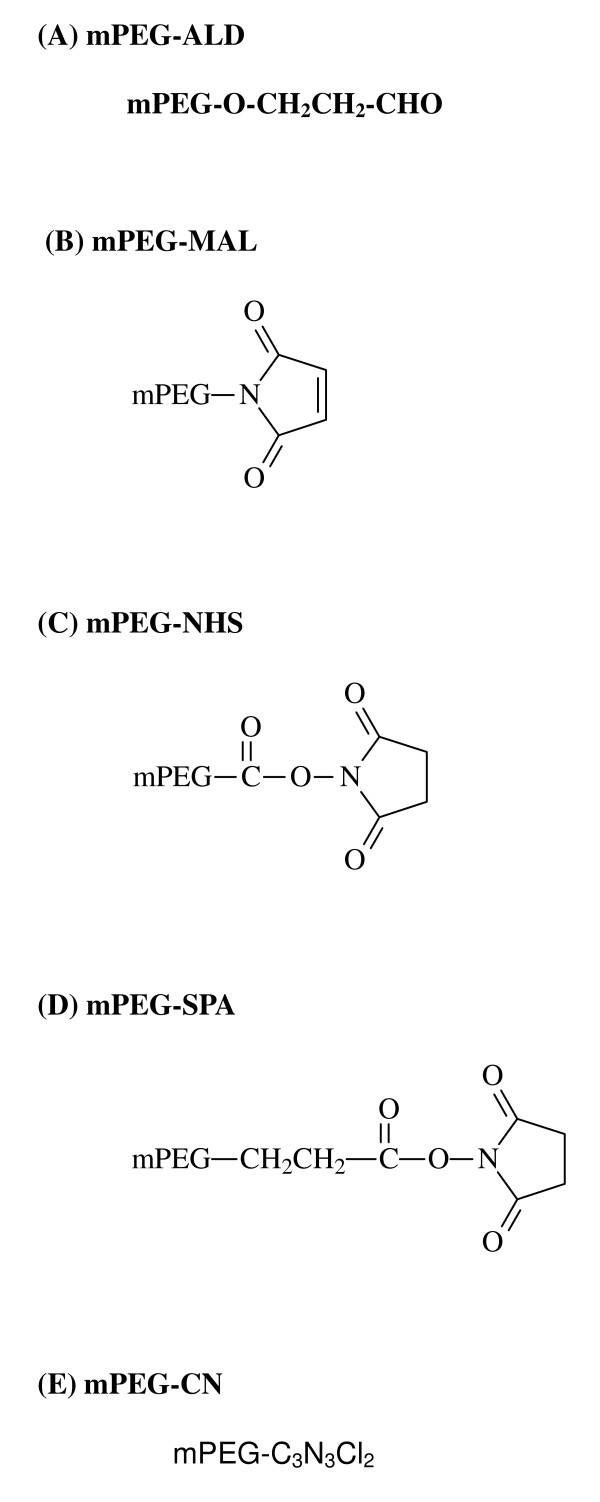
**Chemical structures (molecular formula) of the 5 PEG derivatives used**. (A) mPEG-ALD (methoxypolyethylene glycol-propionaldehyde); (B) mPEG-MAL (methoxypoly-ethylene glycol-maleimide); (C) mPEG-NHS (methoxypolyethylene glycol-N-hydroxy-succinimide); (D) mPEG-SPA (methoxypolyethylene glycol-succinimidyl propionate). (E) mPEG-CN (methoxypolyethylene glycol-cyanuric chloride). MW = 5,000 in all cases.

Five different pegylated forms of arginase 1 have been examined (Fig. 1). We briefly report on their pharmacodynamics and general effects on a variety of blood and body parameters, which show the relative absence of side effects. In all, the following aspects have been taken into account: (1) determination of the most appropriate type of PEG molecule to use; (2) optimization of the conditions for the pegylation reaction, (3) the effects of pegylation on enzyme activity, (4) the effectiveness of the pegylated enzyme to deplete arginine *in vivo *(in rats), and (5) the sensitivity of two human hepatocellular carcinoma (HCC) cell lines in vitro to these pegylated molecules. We found that conjugation of rhArg1 with mPEG-SPA 5,000 proved most effective on all these accounts, having already dismissed PEG 20,000 used by workers favouring ADI as the catabolic enzyme of choice [5,22].

## Results

### Pegylation of rhArg1

**Figure 2:**
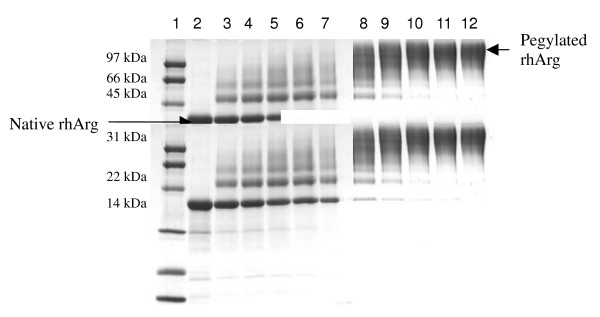
**SDS-PAGE (15% gel) analysis of native and mPEG-SPA pegylated rhArg11**. Time-course of the pegylation reaction with 1:20 and 1:50 (mol/mol) of rhArg11:mPEG-SPA. Lane 1, low-range protein marker; Lane 2, native rhArg1; Lane 3, 1:20 mole ratio (0.5 h); Lane 4, 1:20 mole ratio (1 h); Lane 5, 1:20 mole ratio (2 h); Lane 6, 1:20 mole ratio (3 h); Lane 7, 1:20 mole ratio (20 h); Lane 8, 1:50 mole ratio (0.5 h); Lane 9, 1:50 mole ratio (1 h); Lane 10, 1:50 mole ratio (2 h); Lane 11, 1:50 mole ratio (3 h); Lane 12, 1:50 mole ratio (20 h).

rhArg1 was expressed in *B. subtilis *as a N-terminal 6×His-tagged protein [4,11]. The enzyme was purified by nickel affinity column chromatography, and ~100 mg of the native rhArg1 (>95% purity) was routinely obtained per liter of culture. Its MW was ~35 kDa when analyzed by SDS-PAGE (lane 2 in Fig. 2).

mPEG-ALD, mPEG-NHS, mPEG-SPA and mPEG-CN are all lysine-active PEGs. The most frequently used derivatives for lysine attachment are the N-hydroxylsuccinimide (NHS) active esters, such as PEG succinimidyl succinate (mPEG-SS) and succinimidyl propionate (mPEG-SPA). Reaction between the epsilon amino group of lysine(s) or the N-terminal amine and the NHS ester produces a physiological stable amide linkage(s). Increasing pH increases the rate of reaction and lowering pH reduces rate of reaction. These highly "active esters" can couple at physiological pH, and low temperature can be used if a labile protein is being conjugated. In this case, a long reaction time is useful. Analysis of several reactions with varying ratios of protein:PEG and at different pHs quickly provide information to help decide the conditions giving the required level of pegylation. Aldehydic preparations, such as mPEG-ALD, are much less reactive than NHS esters. On the other hand, coupling of sulfhydryl-selective PEG such as mPEG-MAL 5,000 and thiol groups is one of the most useful reactions for bioconjugate preparations. This reaction is highly specific and takes place under mild conditions in the presence of other functional groups. mPEG-MAL has been used as a reactive polymer for preparing well-defined bioactive PEG-protein conjugates. The sulfhydryl-selective chemistry of mPEG-MAL 5,000 is in Phase II clinical trials.

rhArg1 was formulated (pegylated) with PEG molecules of various linkers (see also Fig. 1). The relative enzyme activities of the various types of pegylated rhArg1 are shown in Table 1. Pegylation with mPEG-CN caused a dramatic decrease in the enzyme's specific activity, a drop of ~70% relative to the native rhArg1, whereas all other pegylated forms only decreased activity by 10–15%. mPEG-SPA (MW = 5,000) was selected as the most suitable candidate for the pegylation of rhArg1, because the resulting modified enzyme, designated rhArg1-peg_5,000 mw_, retained >90% of the native rhArg1 enzymatic activity (Table 1). In addition, mPEG-SPA does not have an ester linkage in the backbone (Fig. 1), and therefore generates stable linkages, with nearly ideal reactivity for protein modification. mPEG-SPA is being used in Pfizer's SOMAVERT (Pegvisomant for injection) for the treatment of acromegaly (over-expression of human growth hormone) in patients who responded poorly to surgery and/or radiation therapy [23].

**Table 1:** rhArg1 was reacted with five different PEGs, varying in linker chemistry.

Type of PEG used for pegylation	rhArg1: PEG ratio (mol/mol) for pegylation	Relative arginase activity (%)
Native rhArg1	---	100
mPEG-SPA	1:20	94
	1:50	93
mPEG-NHS	1:20	86
	1:50	83
mPEG-MAL	1:20	85
	1:50	84
mPEG-ALD	1:20	88
	1:50	88
mPEG-CN	1:20	34
	1:50	30

Different forms of pegylated rhArg1 were prepared, purified and analyzed for their specific activities. Relative arginase activity indicates the specific activity of pegylated rhArg1 with respect to native rhArg1.

To optimise the reaction time and the amount of mPEG-SPA required for the complete pegylation of rhArg1, the native enzyme was incubated with 1: 20 and 1: 50 mPEG-SPA concentrations for varying periods of time. A rhArg1: PEG molar ratio of 1: 50 was sufficient to ensure undetectable levels of native rhArg1 after 20 h of reaction. However, after 3 h of reaction, the amount of unmodified rhArg1 was already close to undetectable, i.e. a further 17 h did not significantly increase the degree of pegylation (Fig. 2). From these results the optimum rhArg1: PEG ratio and reaction duration were found to be 1: 50 and 3 h, respectively. Under these mild conditions, the other PEGs tested (mPEG-ALD, mPEG-MAL, mPEG-NHS and mPEG-CN) gave only partial pegylation reaction products and different amounts of native rhArg1 were still detected (data not shown).

**Figure 3:**
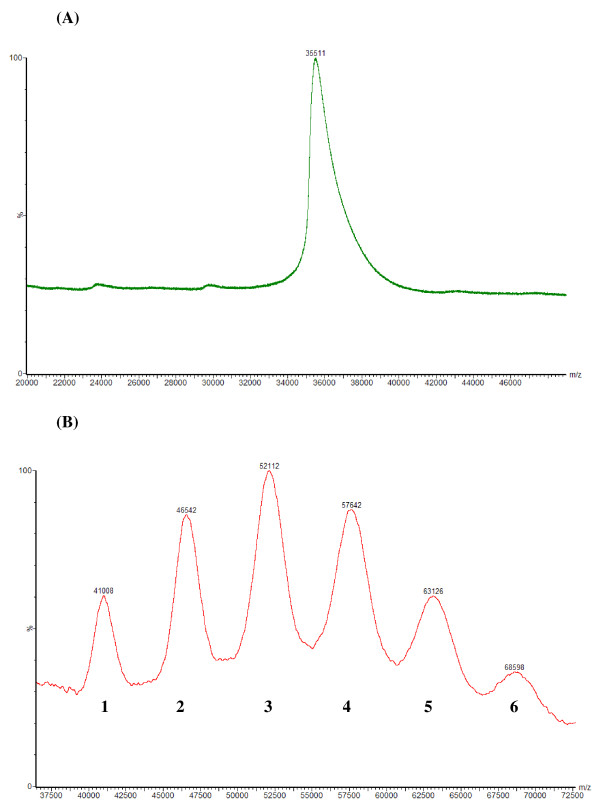
**MALDI-TOF-MS results of native and pegylated rhArg11**. (A) MW of the native rhArg11 is ~35.5 kDa. (B) Numbers 1–6 under the peaks correspond to the number of PEG conjugated; the numbers on the peaks indicate the mass-to-charge (m/z) ratio values.

The pegylation reaction products generated by using mPEG-SPA were analyzed by SDS-PAGE (Fig. 2), which indicates that the MW of rhArg1 was increased after conjugation with mPEG-SPA. MALDI-TOF mass spectra of native rhArg1 and PEG-conjugated rhArg1 are shown in Fig. 3. Based on amino acid sequence, the calculated MW of native rhArg1 is 35,558, consistent with the measured MW of 35,511 (Fig. 3A). The spectrum of pegylated rhArg1 has 6 peaks, labeled 1–6 in Fig. 3B, with m/z values of 41,008, 46,542, 52,112, 57,642, 63,126 and 68,598, respectively. The number shown at the bottom of each peak in Fig. 3B corresponds to the number of PEG conjugated to rhArg1. The MW difference between adjacent peaks in the spectrum is ~5,000, consistent with the average MW of the PEG used for the pegylation of rhArg1. Polydispersity is a ratio that represents the broadness of a molecular weight distribution. If the polydispersity is equal to 1, the polymer is said to be monodispersed; typically, however, polymers are not truly monodispersed. Therefore, we conclude that rhArg1-peg_5,000 mw _has 1–6 molecules of PEG attached to the free amino groups of Lys residues in arginase 1.

A pegylated protein is typically a mixture of varying numbers of PEGs of different molecular weights attached to several amino acids. Each starting PEG sample used to prepare a particular reagent contains a range of PEGs of varying molecular weight (typical polydispersity values are <1.05), hence proteins containing the same number of PEGs will have a range of molecular weights. These PEGs will be distributed on different amino acids.

### Enzyme kinetic analysis

The K_m _value of native rhArg1 for the substrate arginine was 1.9 ± 0.7 mM, which is similar to the K_m _values of native human liver arginase (2.6 mM, [24]) and human erythrocyte arginase (1.6 mM, [20]) for arginine. Intriguingly, the K_m _of pegylated rhArg1 (rhArg1-peg_5,000 mw_) was found to be ~2.9 mM, indicating that the attached PEG molecules on the surface of the enzyme did not dramatically reduce the affinity for the substrate. These results are at variance with those reported for pegylated bovine liver arginase [19,25].

Arginase converts arginine to ornithine and urea. Despite its strong *in vitro *anti-cancer properties, the bovine liver arginase has never been seriously considered as a potential drug for the treatment of human cancers for several reasons, including its much lower affinity for arginine (*K*_m_, 6 mM for the native enzyme; *K*_m_, 12 mM for the pegylated enzyme) at physiologic pH [25], a pH optimum of 9.6, and a short circulatory half-life (a few minutes). These have been considered serious shortcomings since the 1980s, with the report from Savoca et al. [19] showing no antitumor activity of bovine liver arginase in mice bearing the Taper liver cancer. Other investigators have also given negative reports on the bovine arginase [6]. It appears that the bovine enzyme has quite different biochemical properties compared with human liver arginase.

### CD spectroscopic analysis

**Figure 4:**
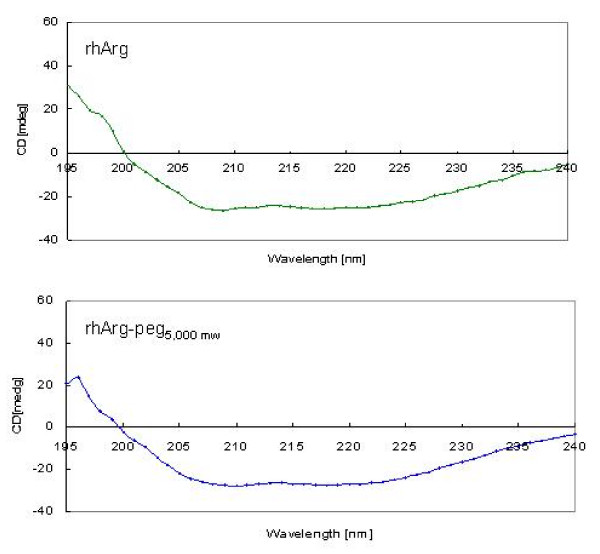
**CD spectroscopic analysis**. CD spectra (195–240 nm) of rhArg11 and rhArg11-peg_5,000 mw _under at 0.3 mg/ml in 10 mM potassium phosphate buffer, pH 7.0, analysed in a Jasco J-810 spectropolarimeter.

The changes in the secondary structures of rhArg1-peg_5,000 mw _compared to native rhArg1 were examined by CD spectroscopy in the far-UV region of the spectra covering the wavelength range 190–250 nm (CD spectra shown in Fig. 4). The spectra of 2 enzymes both exhibited a negative band (magnetic dipole) that is at ~200–240 nm and a positive band (electric dipole) at ~195 nm.

A summary of the CD experimental data with regard to the secondary structural compositions of rhArg1 and rhArg1-peg_5,000 mw _(Table 2) strongly suggests that the pegylated rhArg1 has no significant difference in secondary structure from the native rhArg1 since the percentage composition of each class of structure only varied by a maximum of 4% between the native and pegylated arginases, consistent with the results showing that their enzyme activities were similar (Table 1). Nor does pegylation seem to prevent the access of arginine to the active site, which is not unexpected given the small size of the substrate.

**Table 2:** Secondary structure compositions of native and pegylated rhArg1 estimated by using CD analysis software.

	Native rhArg1	rhArg1-peg_5,000 mw_	Native rat liver arginase
α-Helix	25%	24%	22%
β-Sheet	27%	28%	28%
Turns	13%	17%	17%
Random structure	36%	32%	33%

### Pharmacodynamic studies

**Figure 5:**
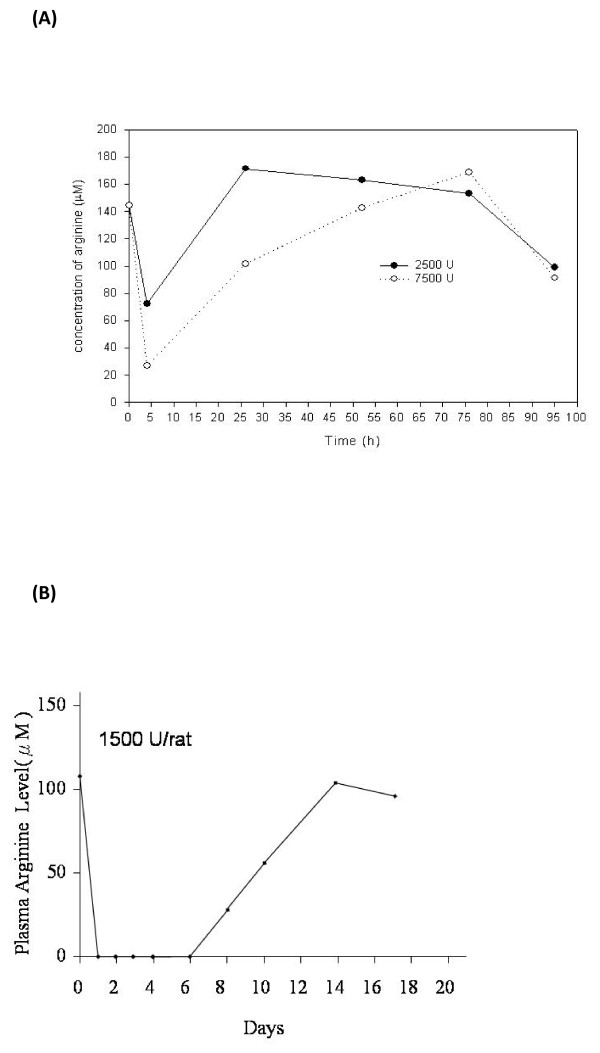
**Pharmacodynamics of (A) native rhArg1 and (B) pegylated rhArg1 on plasma arginine in rats**. Sprague-Dawley rats were each injected i.p. with native or pegylated rhArg11. Plasma was collected at the indicated times, and arginine in each sample was determined by amino acid analysis. The data given as the means and 1 SD of 8 animals.

The *in vivo *studies involved the ip injection of rhArg1 or rhArg1-peg_5,000 mw _into rats, whose plasma arginine levels were measured. The results (Fig. 5B) show that complete arginine depletion was achieved with 1,500 U of pegylated rhArg1 per rat (6 U/g of rat) for 6 days. In comparison, the highest dose of native rhArg1 (7,500 U/rat) only reduced circulating arginine to ~20 μM within 3 h of injection, with arginine levels returning to normal ~2 days post-injection (Fig. 5A).

Ensor et al. [7] found that the circulation half-life of ADI (a few hours) was prolonged by pegylation. The pegylated ADI had a circulation half-life of 2–3 days. Although the native ADI enzyme and the pegylated ADI were similar in their ability to inhibit the growth of melanoma and HCCs *in vitro*, only the pegylated ADI was effective in inhibiting growth of these tumors *in vivo*. To achieve efficacy *in vivo*, serum arginine levels must be maintained at low levels for a sufficient time, and certainly at least 3 days.

### Hematological and biochemical changes during arginine depletion

Most of the hematological, biochemical, and clinical parameters were not significantly different between the control and test groups. The relative organ weights are summarized in Table 3, with only the brain showing for some unknown reason a statistically significant change between the control and treated groups.

**Table 3:** Hematological values, clinical biochemical findings, and organ weights in SD rats after weekly injection of rhArg1-peg_5,000 mw _for 4 weeks.

**Parameters**	**Control**	**15,000 U/kg**	**50,000 U/kg**
**Hematological values**			
RBC (× 10^12^/l)	8.25 ± 0.87	8.91 ± 0.90	8.20 ± 0.89
WBC (× 10^9^/l)	5.27 ± 1.14	5.68 ± 1.08	4.02 ± 0.71*
Hb (g/l)	136.33 ± 17.25	134.50 ± 6.83	122.33 ± 6.80
MCH (pg)	16.50 ± 1.05	15.23 ± 1.67	15.00 ± 1.12*
MCHC (g/l)	342.00 ± 13.31	287.17 ± 35.82**	305.17 ± 19.26**
PLT (× 10^9^/l)	742.50 ± 156.23	663.00 ± 121.51	545.83 ± 113.26*
HCT (%)	0.399 ± 0.05	0.474 ± 0.06*	0.403 ± 0.039
MCV (fl)	48.33 ± 2.25	53.18 ± 4.03*	49.17 ± 0.96
**Clinical biochemical findings**			
ALT (nmol/l)	818.50 ± 69.16	796.83 ± 110.52	733.48 ± 118.66
CK (μmol/l)	112.60 ± 37.41	88.30 ± 27.17	88.14 ± 24.33
CH (mmol/l)	3.219 ± 0.51	2.500 ± 0.6**	2.879 ± 0.639
AST (nmol/l)	2883.90 ± 602.99	2622.20 ± 420.55	2300.46 ± 512.72
ALB (g/l)	42.60 ± 8.38	40.10 ± 18.83	35.86 ± 5.46
TP (g/l)	86.70 ± 12.49	76.20 ± 10.25	84.86 ± 15.08
BUN (mmol/l)	7.919 ± 3.267	9.305 ± 1.200	9.801 ± 0.815
TG (mmol/l)	3.129 ± 1.087	2.672 ± 1.175	2.832 ± 0.112
**Organ weights (g)**			
Brain	0.380 ± 0.063	0.345 ± 0.050	0.324 ± 0.031*
Gland	0.071 ± 0.026	0.080 ± 0.037	0.074 ± 0.023
Heart	0.184 ± 0.021	0.180 ± 0.031	0.195 ± 0.020
Lung	0.237 ± 0.040	0.216 ± 0.051	0.233 ± 0.038

Notes: All values are expressed as mean ± standard deviation. RBC, red blood cell count; WBC, white blood cell count; Hb, hemoglobin; MCH, mean corpuscular hemoglobin; MCHC, mean corpuscular hemoglobin concentration; PLT, platelet count; HCT, hematocrit; MCV, mean corpuscular volume; ALT, alanine aminotransferase; CK, creatine kinase; CH, cholesterol; AST, aspartate aminotransferase; ALB, albumin; TP, total plasma protein; BUN, urea nitrogen in blood; and TG, triglycerides. Significant differences as compared with control by Duncan's *t*-test after ANOVA test (*p < 0.05; **p < 0.01).

### In vitro anti-tumor activity

To compare the anti-tumor efficacies of rhArg1 and rhArg1-peg_5,000 mw_, we applied the enzymes at a range of concentrations in the culture medium of human HCC cell lines (HepG2 and Hep3B). IC_50 _values (i.e. concentrations achieving a 50% inhibition of cell growth) of rhArg1-peg_5,000 mw _were determined at 0.2 and 0.1 U/ml in HepG2 and Hep3B cells, respectively, which is close to the results with native rhArg1 (Table 4). The results demonstrated that pegylated rhArg1 still has high anti-tumor efficacy comparable to the native enzyme.

**Table 4:** IC_50 _values of rhArg1 versus rhArg1-peg_5,000 mw _for human hepatocellular carcinomas *in vitro*.

	rhArg1 (U/ml)	rhArg1-peg_5,000 mw _(U/ml)
HepG2	0.18 ± 0.06	0.22 ± 0.03
Hep3B	0.07 ± 0.03	0.10 ± 0.04

HepG2 and Hep3B were incubated in arginine-containing DMEM medium supplemented with 10% FBS with rhArg or rhArg1-peg_5,000 mw _(0–100 U/ml) for 72 h at 37°C/5% CO_2_. IC_50 _values were calculated as the concentration of native rhArg1 or pegylated rhArg1 which inhibited 50% cell growth. The experiments were repeated three times.

## Discussion

Pegylation greatly improves the stability and solubility, prolongs the circulation half-life, and lowers the immunogenicity of proteins. However, enzyme activity is commonly affected by the process. For example, only a trace biological activity remained in granulocyte colony-stimulating factor when <10% of the primary amino groups were attached to PEG [26]. In contrast, tumor necrosis factor-α as well as ADI retained approximately half of its biological activity when ~40–50% of its primary amines were conjugated with PEG molecules [21,27]. We set out to determine the effects of pegylation on the activity of arginase – a urea cycle enzyme that can potentially be used in anti-cancer treatment [2,4,28] – in an attempt to improve its therapeutic properties.

A wide array of pegylation reagents are available. The NHS esters of PEG carboxylic acids are the most popular derivatives for coupling PEG to proteins. Reaction between lysine and terminal amines and the active esters produces a stable amide linkage. PEG succinimidyl succinate (mPEG-SS) is one of the oldest and most used PEG derivatives, but it possesses an ester linkage in its backbone and thus has the property of undergoing hydrolysis *in vivo*. For example, the ADI-PEGs studied by Holtsberg et al. [21] were synthesized from succinimidyl succinate PEGs, with PEG molecular weights of 5000 Da (ADI-SS PEG_5000 mw_) and 20,000 Da (ADI-SS PEG_20,000 mw_). These ADI-PEGs retained ~50% of enzyme activity when PEG was covalently attached to ~40% of the primary amines irrespective of the PEG molecular weight.

The amine pegylating reagent mPEG-SPA does not have an ester linkage in the backbone, they therefore generate stable linkages, and they have nearly ideal reactivity for protein modification. Moreover, mPEG-SPA 5,000 has been used in several human applications. For example, it has been used for attachment to a protein antagonist, which has successfully completed clinical trials and an NDA has been filed [23]. Therefore, the mPEG-SPA 5,000 process is validated and suitable for commercial use. In the present study, the isolated and purified rhArg1 was covalently attached via a succinamide propionic acid (SPA) linker to polyethylene glycol (PEG) of MW 5,000. Unlike many other pegylated enzymes, this pegylated rhArg1 (rhArg1-peg_5,000 mw_) was fully active (Table 1). The *K*_m _value for arginine as the substrate of the pegylated rhArg1 was also much smaller (*K*_m _= 2.9 mM) than that of the previously reported pegylated bovine liver arginase (*K*_m _= 12 mM) (Savoca et al., 1979). Subsequently, the pegylated rhArg1 was found to be stable enough *in vivo *to maintain sufficient enzyme catalytic activity at physiologic pH [4].

Reaction conditions for coupling PEG to a protein vary depending on the protein, the desired degree of pegylation and the PEG derivative utilized. Factors involved in choice of derivative are: desired point of attachment (lysine vs cysteine); hydrolytic stability and reactivity of the derivative; stability of the linkage; and suitability for analysis. Conjugation of rhArg1 with PEG molecules of various linkers (Fig. 1), and measurement of the relative activities of the various types of pegylated rhArg1 (Table 1), showed that in most cases, pegylation had minimal effects on the activity of rhArg1. Only the conjugation with mPEG-CN produced a significant negative effect on the enzyme's specific activity, which exhibited a drop of ~70% relative to the native rhArg1, whereas all the other modifications only decreased the activities by ~10–15%. The rhArg1 conjugated with mPEG-SPA (MW = 5000), designated rhArg1-peg_5,000 mw_, retained >90% of the native enzymatic activity, and was thus selected for further studies. The observed 10% drop in activity of the rhArg1-peg_5,000 mw _is favorable when compared to numerous other proteins, such as ADI, granulocyte colony-stimulating factor (GCSF) and TNF-α, where pegylation normally results in a 30–70% activity loss [21,26,27]. In the case of mPEG-CN, not only was it once the most widely used reagent for protein pegylation, it was also successfully used in the pegylation of bovine liver arginase in 1979 [25]. Despite this, our mPEG-CN pegylated rhArg1 had the lowest specific activity, retaining only ~30% of its native activity (Table 1). This decrease in specific activity after pegylation may be due to steric clash between the PEG molecules and rhArg1's substrate which obstructs substrate binding.

mPEG-NHS and mPEG-ALD were both also unsuitable for the pegylation of rhArg1 as they did not sufficiently react with the native enzyme, as shown by a large amount of unpegylated rhArg1 in SDS-PAGE analysis (data not shown). A possible explanation for this low yield is that the mild pegylation conditions (in PBS buffer, pH 7.4, 25°C) were not suitable for the coupling of mPEG-NHS and mPEG-ALD to arginase. Since the pegylated rhArg1 is going to be targeted for therapeutic use, severe pegylation conditions should be avoided as much as possible. mPEG-MAL was also found to be an improper candidate due to the relatively small size (low MW) of the pegylated enzyme, which is a disadvantage as it is unlikely to be sufficiently protected from degradation and would still have a high rate of renal clearance [26]. In fact, there is a correlation between the molecular size of the pegylated protein and its circulatory half-life: the radii of pegylated protein molecules are inversely related to the rate of renal clearance [18]. As a consequence the half-life of the pegylated protein would not be significantly prolonged. Therefore, out of the 5 PEG molecules tested, our results showed mPEG-SPA to be by far the most promising candidate for further studies concerning the production of a therapeutic-grade pegylated rhArg1. Based on our results, the optimum rhArg1:PEG ratio and reaction duration were 1:50 and 3 h, respectively.

Gel electrophoresis (SDS-PAGE) does not provide quantitative information because PEG "acts" much larger than the proteins used for calibration (Fig. 2). However, this sizing technique is extremely useful for qualitative analysis of PEG proteins because it demonstrates the occurrence of pegylation and it is very effective in monitoring reproducibility of preparations. Determination of overall heterogeneity in a PEG-protein (i.e., number of PEG-protein forms present) remains difficult and is certainly not a routine matter. One of the most significant recent advances in PEG and PEG-protein analysis has been the availability of MALDI-TOF-MS. This technology provides the mass of the unfragmented, singly-charged molecular ion of macromolecules up to about 100,000 Da, and thus greatly assists determination of polydispersity and identity of PEG derivatives. Similarly, the mass of the molecular ions of PEG conjugates, such as PEG-proteins, can also be determined, and the composition of pegylation reaction products containing different numbers of PEGs ("one-mer", "two-mer", etc.) can be established. As shown in Fig. 3, rhArg1-peg_5,000 mw _has 1–6 molecules of PEG attached to the amines of Lys residues of the arginase, and under the reaction conditions we employed, the major isotypes include the "two-mer", "three-mer" and "four-mer".

The K_m _value of rhArg1 for arginine was 1.9 ± 0.7 mM. However, the K_m _of the pegylated enzyme increased by ~1 mM, giving it a significantly higher K_m _when compared to ADI, which has a K_m _of ~0.1–0.2 mM [28]. Although the K_m _value of the pegylated rhArg1 seems to be rather high to deplete efficiently plasma arginine (normal range ~100–150 μM) [28], it might be considered that this enzyme would be incapable of depleting systemic arginine levels unless it was present for a considerable period of time at high dose levels, in particular when compared with the biochemical attributes of ADI. Importantly, pegylation of rhArg1 with mPEG-SPA lengthened the depletion period of serum arginine to one that is therapeutically acceptable, and sufficiently long to compensate for an apparent loss in activity, so that large daily doses would no longer have to be given to patients in order to achieve and maintain arginine depletion. It is also noteworthy that the V_max _of arginase compensates in very good measure for its lower affinity, largely canceling out the much lower K_m _for ADI.

The *in vivo *studies with rats confirmed the efficiency of rhArg1-peg_5,000 mw _by showing that a single dose of 1,500 U was capable of depleting serum arginine levels for up to 6 days after the pegylated enzyme was administered (Fig. 5B). Intake of rhArg1-peg_5,000 mw _up to 4 weeks did not produce any apparent ill effects in these animals, which continued to gain weight with no behavioral changes. Their blood, liver and renal chemistries, platelet counts and clotting studies were normal throughout the study (Table 3). This is consistent with the reported findings in the pegylated ADI study, which is now in phase III clinical development in Italy and MD Anderson Cancer Center in Texas [22,29]. Our data are also consistent with the fact that the mPEG-SPA advanced pegylation reagent is a clinically proven stable attachment chemistry, which enables improved therapeutic safety and efficacy and decreased dosing frequency [23].

In **conclusion**, the pegylation of rhArg1 with mPEG-SPA (MW 5,000) significantly prolonged the duration of arginine depletion while retaining >90% of its activity, and this has been shown to be sufficient to offset the low activity of rhArg1 under physiological conditions, allowing sustained arginine depletion to be achieved *in vivo*. We also believed that the enzyme's half-life has been prolonged after pegylation, but it will need further elucidation. Notably, PEG conjugation of rhArg1 still maintains its tumor-specific efficacy (Table 4). Furthermore, we have already shown that the growth of the ADI-resistant Hep3B tumor cells in mice was inhibited by treatment with pegylated rhArg1 (rhArg1-peg_5,000 mw_), which is active alone and is synergistic in combination with 5-fluorouracil [4]. By broadening our knowledge of the biological and pharmacological characteristics of both the rhArg1 and pegylated rhArg1, the present studies will make it possible to facilitate the development of arginase as pharmacological agents in the treatment of human cancer by arginine deprivation. Treatment of human cancer (HCC) patients with the chosen pegylated arginase has been given on compassionate grounds, with consent. We are therefore assured that the drug is safe to use in cancer clinics. We believe that arginase (generally arginine depletion) is going to be a significant force in cancer therapy whether applied alone or in combination with other drugs.

## Methods

### Pegylation of recombinant human arginase (rhArg1)

rhArg1 was obtained by producing the 6×His-tagged human arginase I (liver arginase) enzyme in *Bacillus subtilis *[11]. The protein was pegylated in separate reactions with the 5-methoxypolyethylene glycol (mPEG) of MW 5,000 (Fig. 1). mPEG-ALD, mPEG-MAL, mPEG-NHS, and mPEG-SPA were purchased from Nektar Therapeutics (Huntsville, AL, USA), and mPEG-CN from Sigma (St. Louis, MO, USA). mPEG-ALD is readily oxidized by atmospheric oxygen to produce the corresponding carboxylic acid, making storage under nitrogen essential for this reagent, and thus points immediately to its. Derivatives such as the succinimidyl active esters (mPEG-SPA and mPEG-NHS) are reactive with moisture, and therefore are stored in an argon atmosphere at -20°C.

Each type of PEG was conjugated with rhArg1 in PBS buffer (pH 7.4) at a protein:PEG molar ratio of 1:20 or 1:50. Samples were stirred at 25°C for 3 h to allow sufficient PEG molecules to react with rhArg1. The extent of rhArg1 pegylation was analyzed by carrying out SDS-PAGE, and protein concentration was determined by the Bradford assay (Bio-Rad, Philadelphia, PA, USA). The resulting products were exhaustively dialyzed against PBS buffer (pH 7.4) using an FX 50 s High-Flux Dialyzer (Fresenius Polysulfone, Germany) in order to remove unbound PEG molecules, which were stored at 4°C.

### Enzyme activity and kinetic analysis of native and pegylated rhArg1

A coupled spectrophotometric assay, following the decrease in absorbance at 340 nm (A_340_), was used to determine arginase activity [12]. The specific activity of the purified native rhArg1 was ~400 U/mg protein, 1 U of arginase being defined as the amount of enzyme that can produce 1 μmol urea/min at 30°C, pH 8.5.

To obtain kinetic data, including K_m _values, of the native and pegylated rhArg1, the concentration of arginine was varied from 0 to 50 mM and the corresponding changes in A_340 _values were measured. The initial velocity data were fitted with non-linear regression plots using Stanislawski's program (Trinity Software, Plymouth, USA) to calculate K_m _values.

### Determination of the degree of pegylation by MALDI-TOF-MS

PEG attached to one or more of several potential sites – lysine and N-terminal amino groups – on the enzyme, each attachment location defining a different isotype. The distribution of PEG isotypes has interesting implications in drug development process, which is because the product must be defined by the distribution specification of conjugation sites, i.e. the activity of the product is a function of the defined mixture.

The number of mPEG-SPA molecules attached to the primary amines of rhArg1 was determined by MALDI-TOF-MS. Mass spectra were acquired using a Waters^® ^Micromass^® ^MALDI micro MX™ Mass Spectrometer. Ions were generated by a nitrogen laser operating at a wavelength of 337 nm. Data were acquired in the positive linear mode of operation. The accelerating voltage in the ion source was 18 kV and the extraction delay 500 ns. The matrix, 10 mg/ml spinapinic acid, solution was freshly prepared in 50% of 0.1% aqueous trifluoroacetic acid and 50% acetonitrile. Equal volume of matrix solution was mixed with the protein sample and 1.5 μl of the solution spotted on a stainless steel sample plate. The mixture was allowed to air dry before being introduced into the mass spectrometer.

### Protein secondary structure analysis

Circular dichroism (CD) was used to identify any significant changes in the secondary structure of rhArg1 after pegylation. By measuring the CD spectra between 190 and 250 nm, the relative abundance of the various secondary structural types were resolved [30,31], making it possible to detect significant structural changes in the pegylated enzyme. Native or pegylated rhArg1 (0.3 mg/ml) in 10 mM potassium phosphate (pH 7.0) was measured, using a Jasco J-810 spectropolarimeter (Great Dunmow, UK) at 25°C using cuvettes of 1 mm path length. Each CD spectrum was the average of 2 scans and the CD signals were expressed as mdeg.

### Pharmacodynamic studies

Sprague-Dawley (SD) rats from the Chinese University of Hong Kong (Shatin, Hong Kong) were used in the pharmacodynamic studies. Normal SD rats (4 females and 4 males) of 3 months of age (average weight ~250 g) were randomly assigned into 3 groups. Native rhArg1 (2,500 and 7,500 U per rat) or rhArg1-peg_5,000 mw _(1,500 U per rat) were given i.p. on day 0. Blood samples were drawn from their tail veins on day 0 before injection, and on days 1 to 6, and thereafter every 2 days. Blood was collected in EDTA and mixed with 50% trichloroacetic acid for precipitation of protein by incubating on ice for 30 min. The samples were centrifuged at 13,000 × *g *for 15 min, and arginine level in the clear supernatant fraction was analyzed by high-speed amino acid analyzer (model L-8800, Hitachi). The plasma arginine levels at the indicated time points were determined using the amino acid analyzer as described by Cheng et al. [3].

### Assessment of hematological and biochemical changes during arginine depletion in rats

Three groups of normal SD rats (40 per group) were given saline, 15,000 U/kg, and 50,000 U/kg of rhArg1-peg_5,000 mw _once per week. The rats in each group was killed after 4 weeks and venous blood samples were taken to measure hematological parameters such as hemoglobin, WBC, platelet count, clotting studies (prothrombin time and activated partial thromboplastin time), and full renal and liver chemistries.

### Inhibition of in vitro cancer cell proliferation

Liver cancer cell lines, HepG2 (HBV-negative hepatoblastoma, HB-8065) and Hep3B (HBsAg-positive HCC, HB-8064), from American Type Culture Collection (Manassas, USA), were grown in DMEM complete medium (Life Technologies, Paisley, England) at 37°C in a humidified atmosphere of 5% CO_2 _in air. Cells were seeded at ~2.5 × 10^3 ^cells per well in 96-well plates and allowed to settle overnight. The stated concentrations of rhArg1 and rhArg1-peg_5,000 mw _were diluted into the growth medium and the plates were incubated for 3 days. Quantitative cell proliferation assays were performed using the MTS reagent [3-(4,5-dimethylthiazol-2-yl)-5-(3-carboxymethoxyphenyl)-2-(4-sulfophenyl)-2H-tetrazolium] according to the instructions provided by the manufacturer (Promega, Madison, USA).

## Abbreviations

PBS: phosphate buffered saline; MW: molecular weight; SDS-PAGE: polyacrylamide gel electrophoresis in sodium dodecyl sulphate; MALDI-TOF-MS: matrix-assisted-laser-desorption-ionization-time-of-flight mass spectrometry; ADI: arginine deiminase; PEG: polyethylene glycol.

## Competing interests

PNMC and DNW have interests in Bio-Cancer Treatments International. There are no other competing interests.

## Authors' contributions

Y-CL and W-HL conceived of the study and supervised the project along with DNW, the latter also preparing the final English version of the paper. All the other authors contributed according to their specialists skills in chemistry, molecular biology and biochemistry, and cell biology.
